# Effect of age on exercise capacity and cardiac reserve in patients with pulmonary atresia with intact ventricular septum after biventricular repair

**DOI:** 10.1186/1532-429X-14-S1-P123

**Published:** 2012-02-01

**Authors:** Soha Romeih, Maarten Groenink, Mart N van der Plas, Nico A Blom, Barbara J Mulder, Anje M Spijkerboer

**Affiliations:** 1 Cardiology, Academic Medical Center, Amsterdam, Netherlands; 2 Radiology, Academic Medical Center, Amsterdam, Netherlands; 3 Pulmonology, Academic Medical Center, Amsterdam, Netherlands; 4 Pediatric Cardiology, Academic Medical Center, Amsterdam, Netherlands

## Summary

Pulmonary atresia with intact ventricular septum (PAIVS) is a rare type of congenital heart disease. Biventricular surgical repair is considered to be the optimal treatment option as it provides satisfactory results in terms of survival and clinical outcome during the early follow up period. However the long term clinical fellow-up studies are limited. In the present study we evaluated the cardiac response to the physical and pharmacological stress using dobutamine stress MRI in children and young adults with PAIVS after biventricular repair.

## Background

In patients with pulmonary atresia with intact ventricular septum (PAIVS) biventricular repair is considered to be the optimal treatment option in the absence of significant right ventricular (RV) hypoplasia. However, long term clinical outcome studies are limited. We evaluated exercise capacity and cardiac function during pharmacological stress in children and young adults with PAIVS after biventricular repair.

## Methods

Ten PAIVS patients after biventricular repair, median age 12 years (range 9 - 42 years), underwent a cardiopulmonary exercise test, dobutamine stress magnetic resonance imaging (MRI) and delayed contrast enhancement (DCE) MRI.

## Results

**Figure 1:**
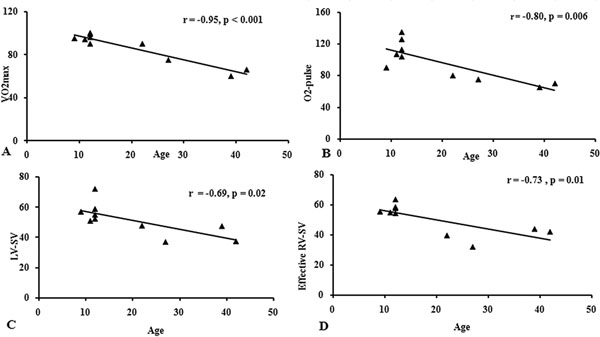
Correlation between the patients’ age and cardiac work indices in response to the physical stress (A) VO2max, (B) O2-pulse. Correlation between the patients' age and biventricular SV response to the pharmacological stress; (C) LV-SV, (D) effective RV SV.

**Figure 2:**
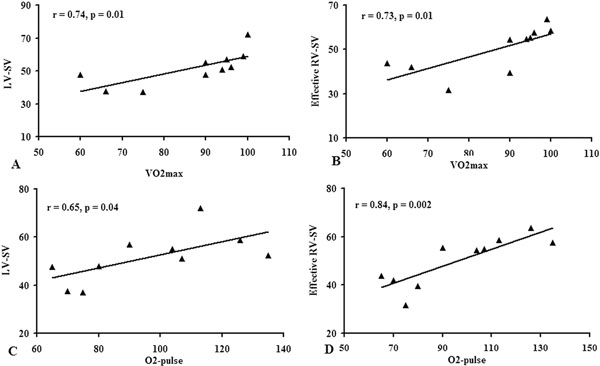
Correlation between VO2max and biventricular SV response to the pharmacological stress; (A) LV-SV (B) effective RV-SV. Correlation between O2-pulse and biventricular SV response to the pharmacological stress; (C) LV-SV (D) effective RV-SV.

The patients' age negatively correlated with exercise capacity (r = -0.95, p = < 0.001) as well as left and right ventricular stroke volume (SV) response to pharmacological stress (r = -0.69, p = 0.02; r = -0.73, p = 0.01, respectively- Figure 1). Furthermore, older age was associated with decreased RV E/A volume ratio and increased pulmonary late diastolic forward flow percentage (r = -0.70, p = 0.02 and r = - 0.80, p = 0.005, respectively) RV E/A volume ratio positively correlated with RV-SV response to DS-MRI (r = 0.77, p = 0.009). VO2max and O2-pulse during physical stress correlated with biventricular SV response to DS-MRI (Figure 2). No right or left ventricular myocardial fibrosis was detected.

## Conclusions

In PAIVS patients after biventricular repair exercise capacity and cardiac reserve decrease with age. These findings appear to be related to impaired diastolic RV function and decreased RV filling, indicating that the function of the relatively small RV deteriorates with time.

## Funding

Financial disclosure: none.

